# Influence of Ionizing Radiation on Fluoride-Releasing Dental Restorative Materials

**DOI:** 10.3390/polym15030632

**Published:** 2023-01-26

**Authors:** Sarah Turjanski, Matej Par, Lana Bergman, Majana Soče, Timor Grego, Eva Klarić Sever

**Affiliations:** 1 Department of Pedodontics, School of Dental Medicine, University of Zagreb, Gunduliceva 5, 10000 Zagreb, Croatia; 2 Department of Endodontics and Restorative Dentistry, School of Dental Medicine, University of Zagreb, Gunduliceva 5, 10000 Zagreb, Croatia; 3 Department of Prosthodontics, School of Dental Medicine, University of Zagreb, Gunduliceva 5, 10000 Zagreb, Croatia; 4 Department of Oncology, Radiotherapy Unit, University Hospital Centre Zagreb, 10000 Zagreb, Croatia

**Keywords:** radiotherapy, dental materials, alkasite, glass hybrid, microhardness, surface roughness, flexural strength, flexural modulus, material reliability, Fourier transform infrared spectroscopy

## Abstract

This study aimed to investigate the effects of radiotherapy on the mechanical, chemical, and surface properties of two recently introduced restorative dental materials (a glass hybrid and an alkasite), while two conventional restorative materials served as references. Material specimens of the experimental groups (irradiated) were compared to the specimens of the control groups that underwent the same preparation procedure but without irradiation. The experimental groups of restorative material specimens were irradiated with a total of 70 Gy over 35 days (2 Gy/day × 35 days), while the control groups received no treatment. The following properties were evaluated: surface microhardness (Vickers), surface roughness, color change, flexural strength, flexural modulus, material reliability, and infrared spectra. For the experimental groups, measurements were performed 24 h after specimen preparation, i.e., before radiotherapy and after the completion of the irradiation protocol. For the control groups, measurements were performed after the corresponding periods of no treatment. A statistically significant increase in microhardness (*p* = 0.001–0.004) and surface roughness (*p* = 0.013) was observed as a result of material aging/maturation in both the control and experimental groups. However, the only statistically significant difference between the control and experimental groups was observed in the discoloration of the conventional reference material (*p* < 0.001). In conclusion, no statistically significant negative effects of a therapeutic dose of radiotherapy on any of the tested properties of the alkasite and glass hybrid materials were observed, whereas only a minor negative effect of radiotherapy in terms of discoloration was found for a conventional resin composite that was used as a reference material.

## 1. Introduction

Provided that restorative treatment has been carefully performed, the success and ultimate survival of dental restorations depend on the properties of the restorative materials and their interfaces with the dental hard tissues [1]. The most common causes of dental restoration failure are bulk fractures and secondary caries, both of which are due to inherent deficiencies of restorative materials [2]. Bulk fractures are primarily the result of inadequate mechanical properties, while secondary caries is often due to degradation and subsequent leakage around restorations made from resin composites [3].

A dentist can select the most suitable material for a particular indication from a variety of materials with different properties. However, despite numerous improvements in restorative materials over the past decade, all restorative materials are subject to some degree of aging-related degradation [4]. As dental restorations are exposed to the harsh environment of the oral cavity throughout their lifetime and are affected by mechanical, chemical, and thermal factors, it is not surprising that they are subject to multifaceted degradation processes. In the case of resin composites, degradation occurs mainly by enzymatic hydrolysis of the polymer matrix and the interface between glass fillers and the matrix [5]. Glass ionomers are mainly degraded by exposure to acids of endogenous or exogenous origin and by cyclic mechanical loading [6]. While these basic effects are inevitable in any functional dentition, many additional patient-specific factors also play a role in accelerating material degradation [7]. These include the iatrogenic exposure of restorative materials to irradiation as part of head and neck radiation therapy.

The high prevalence of head and neck cancer and the effectiveness of radiotherapy as a primary or adjuvant treatment [8] mean that teeth and dental restorations are frequently exposed to the potentially harmful effects of high-energy radiation. Therapeutic doses of radiation have been shown to exert deleterious effects on the microstructure of dental hard tissue [9], although the clinical implications of the resulting microstructural damage are unclear. While it is indisputable that the longevity of dental restorations is significantly impaired in patients undergoing head and neck radiotherapy [10], it is unclear to what extent their failure is caused directly by radiation on restorative materials and dental hard tissues [11] or indirectly by decreased salivary flow and changes in oral flora associated with radiotherapy [12].

Various effects of therapeutic radiation have been studied, but the results in the literature are inconsistent. Virtually all possible outcomes have been described, ranging from no effect [13,14], a positive effect [15,16,17], or a negative effect [18,19] on various material properties. Since dental restorative materials vary in composition, structure, and properties, the inconsistencies in the reports reflect the heterogeneous responses of the tested materials to radiation. This heterogeneity is compounded by differences in irradiation protocols, such as the use of fractionated irradiation or single dose irradiation. Despite the inconclusive literature reports, a systematic review [20] concluded that the most common outcome was no effects (3/5 studies), while negative effects (1/5 studies) and positive effects (1/5 studies) were also reported but much less frequently. The review, published in 2017, was about the effects of radiotherapy on the mechanical properties of restorative materials based on only five strictly selected articles considering methodological soundness, scientific rigor, and acceptable risk of bias. It should be noted that the authors of this systematic review pointed out that the large methodological heterogeneity of the five studies analyzed precluded a quantitative assessment of their results in a meta-analysis.

Although there is currently no convincing evidence of a negative effect of radiotherapy on commonly used restorative materials, the emergence of new restorative materials with altered compositions and properties requires continued investigation of their sensitivity to radiation. Two recently introduced restorative materials that are particularly suitable for patients undergoing head and neck radiotherapy are the “alkasite” material Cention Forte and the glass hybrid material Equia Forte HT. These two materials are especially appropriate for xerostomic patients undergoing head and neck radiotherapy due to their caries-preventive potential combined with favorable mechanical properties. The alkasite material consists of a methacrylate matrix filled with three different types of glass fillers, namely a conventional inert barium alumino-silicate glass, a calcium barium alumino-fluoro-silicate ionomer glass, and a basic calcium fluoro-silicate glass [21]. The material is capable of releasing calcium and fluoride ions, precipitating hydroxyapatite on its surface, and increasing pH. The glass hybrid is the result of the development of high-strength restorative glass ionomer cement and consists of highly reactive nano-sized fluoro alumino-silicate glass and high-molecular-weight polyacrylic acid [22]. These compositional adjustments enabled an improvement in mechanical properties while maintaining all the advantages of glass ionomer materials, i.e., fluoride release, moisture tolerance, self-adhesiveness, and a durable bond with dentin.

Due to the inherently higher degradation tendency of glass-ionomer-based materials and ion-releasing composites, we investigated whether exposure to ionizing radiation as part of head and neck radiotherapy can accelerate regular aging-related degradation processes. Since the effects of irradiation on the novel alkasite and glass hybrid materials have not been previously investigated, this study aimed to examine how their mechanical, chemical, and surface properties are affected by 70 Gy irradiation. As a null hypothesis, it was assumed that radiotherapy would not affect surface microhardness, surface roughness, color, flexural strength, flexural modulus, material reliability, and infrared spectra.

## 2. Materials and Methods

### 2.1. Restorative Materials

The details of the composition of the restorative materials studied are listed in Table 1. The primary restorative materials of interest were the alkasite Cention Forte (Ivoclar Vivadent, Schaan, Lichtenstein) and the glass hybrid material Equia Forte HT (GC, Tokyo, Japan), while a conventional high-viscosity glass ionomer Fuji IX (GC, Tokyo, Japan) and a conventional resin composite Tetric EvoCeram (Ivoclar Vivadent, Schaan, Lichtenstein) were used as reference materials.

### 2.2. Specimen Preparation

Two experimental groups were formed for each restorative material, designated C (control) and IR (irradiated). For the measurements of microhardness, surface roughness, color change, and Fourier transform infrared spectroscopy (FTIR), a sample size of *n* = 10 per experimental group was used. Disk-shaped specimens with a diameter of 15 mm and a thickness of 1 mm were prepared in Teflon molds. The materials were placed in the molds and flattened by covering both mold openings with polyethylene terephthalate films and pressing them with a smooth glass plate with a force of 20 N. Light-curable methacrylate-based materials (Cention Forte and Tetric EvoCeram) were illuminated from both sides using a LED-curing unit (Bluephase Style; Ivoclar Vivadent, Schaan, Liechtenstein) with a radiant exitance of 1200 mW/cm^2^ and a curing time of 20 s. The radiant exitance of the curing unit was measured with a NIST-referenced UV–vis spectrophotometer system (MARC; BlueLight Analytics, Halifax, Canada). The specimens of the glass-ionomer-based materials (Equia Forte HT and Fuji IX) were mixed in the capsules according to the manufacturer’s recommendations and left in the molds for 10 min to cure by the acid–base reaction.

For flexural strength/modulus testing, a sample size of *n* = 20 per experimental group was used to obtain a sufficient amount of data for the Weibull reliability analysis. Rectangular rods (2 × 2 × 16 mm) were fabricated in silicone molds according to NIST 4877 [23]. The light-curable resin composites were illuminated with Bluephase Style (Ivoclar Vivadent, Schaan, Liechtenstein) in a partially overlapping manner (3 × 20 s) from both sides. Excess material and flashes were removed with a sharp scalpel and P4000 silicon carbide paper (Buehler, Lake Bluff, IL, USA). After visual inspection for defects and air inclusions introduced during material handling, specimens with defects were discarded and replaced with intact specimens.

For all of the specimens, surface polishing was simulated by 3 min of wet grinding with P4000 silicon carbide paper (Buehler, Lake Bluff, IL, USA), followed by 3 min of polishing with a 0.05 µm aluminum oxide suspension on a polishing cloth. The specimens were randomly assigned to groups C and IR during preparation. Each specimen was individually stored in a closed Eppendorf tube (Eppendorf, Hamburg, Germany) filled with 4 mL deionized water in an incubator at 37 °C for 24 h before starting radiotherapy.

### 2.3. Radiotherapy Procedure

Specimens from the IR group were irradiated with a total dose of 70 Gy over 35 days (2 Gy/day × 35 days). The specimens were removed from Eppendorf tubes, carefully blotted onto cellulose pads, and air-dried as described in the ISO 4049 protocol for drying restorative material specimens. The dried specimens were exposed to a daily radiation dose of 2 Gy using the Siemens Primus linear accelerator (Siemens Healthineers AG, Erlangen, Germany) with a radiation beam of 6 MV and a source-to-surface distance of 100 cm. The specimens were placed between two 2 cm-thick plates of solid water (RW3; PTW, Freiburg, Germany) to ensure homogeneous irradiation. While the IR group specimens were irradiated, the C group specimens were only dried and left undisturbed. After completion of the irradiation cycle, samples from both groups were placed back into Eppendorf tubes containing 4 mL of fresh deionized water and placed in an incubator at 37 °C. The irradiation cycle was repeated after 24 h, with an additional dose of 2 Gy administered. The irradiation cycles were repeated daily for 35 days, resulting in a total irradiation dose of 70 Gy.

### 2.4. Surface Microhardness Measurements

A microhardness tester (CSV-10; ESI Prüftechnik GmbH, Wendlingen, Germany) was used with a load of 100 g and a dwell time of 10 s to measure the Vickers microhardness (MH). A diamond pyramid applied to the sample surface was used for measurement. The initial measurements were performed 24 h after specimen preparation, while the final measurements were performed after the completion of the 70 Gy radiotherapy. For light-cured specimens, the surface that was first illuminated with the curing unit was consistently used to perform MH measurements. Five replicates per specimen were performed at different locations within a diameter of 5 mm from the center, from which the mean value was calculated and used as the statistical unit.

### 2.5. Surface Roughness Measurements

A portable roughness tester (Surftest SJ-210; Mitutoyo, Houston, TX, USA) was used to measure the surface roughness parameter Ra. The following instrument settings were used: stylus speed: 0.1 mm/s, stylus force: 4 mN, cut-off length: 0.25 mm, sampling length: 0.8 mm, and the number of sampling lengths: 5. The initial surface roughness was measured 24 h after specimen preparation, while the final measurements were performed after the completion of the 70 Gy radiotherapy. For light-cured specimens, the surface that was first illuminated with the curing unit was consistently used to perform Ra measurements. Three replicates were performed per specimen at different locations within a diameter of 5 mm from the center, and the mean value of the Ra parameter was calculated to be used as the statistical unit.

### 2.6. Color Change Measurements

A probe spectrophotometer (VITA Easyshade III; VITA, Bad Säckingen, Germany) was used with a standardized white background [24] to measure color changes of the specimen surface 24 h after specimen preparation and after the completion of the 70 Gy radiotherapy. Three replicates per specimen were performed, from which mean values for the parameters L*, a*, and b* were calculated. The values of L*, a*, and b* between the two time points were used to calculate the perceptible color difference parameter ΔE* as follows [25]:

(1) $$\Delta E*=\sqrt{\Delta {L*}^{2}+{\Delta a*}^{2}+{\Delta b*}^{2}}\;$$

### 2.7. Flexural Strength and Modulus Measurements

Specimens for flexural strength (FS) and flexural modulus (FM) measurements were analyzed after exposure to 70 Gy radiation (IR group) and the corresponding control treatment (C group). FS and FM were measured by loading the specimens into a universal testing machine (Inspekt Duo 5kN-M; Hegewald & Peschke, Nossen, Germany) until failure according to NIST 4877 using a three-point setup with a support spacing of 12 mm and a crosshead speed of 1 mm/min [23]. During the test, the specimens were immersed in distilled water at room temperature. The following expressions were used to calculate FS and FM:

(2) $$\mathrm{FS}=\frac{3F_{f}l}{2{\mathrm{bh}}^{2}}\;$$

(3) $$\mathrm{FM}=\frac{F_{l}l^{3}}{4{\mathrm{bh}}^{3}y_{l}}\;$$

where F_f_ = force at fracture (N), l = span between supports (mm), b = specimen width (mm), h = specimen height (mm), F_l_ = force at the end of the linear part of the force–deflection diagram (N), and y_l_ = deflection at the end of the linear part of the force–deflection diagram (mm).

### 2.8. Fourier Transform Infrared (FTIR) Spectroscopy

Specimens for the FTIR study were analyzed after exposure to 70 Gy radiation (IR group) and the corresponding control treatment (C group). FTIR spectra were acquired using a Nicolet iS50 spectrometer (Thermo Fisher Scientific, Waltham, MA, USA) in a spectral range of 4000–400 cm^−1^ and a spectral resolution of 4 cm^−1^. The specimens were dried in a vacuum at room temperature for 7 days and fixed on an attenuated total reflectance (ATR) diamond crystal using a special press. The window of the ATR accessory sampled a circular area (d = 2.5 mm) from the center of each specimen. One spectrum per specimen was acquired by averaging 40 consecutive scans.

### 2.9. Statistical Analysis

A power analysis was performed using G*Power (version 3.1) [26] based on data from a preliminary study to estimate the required sample size to detect with a probability higher than 80% a statistically significant difference between the C group and the IR group of at least 15% at a 0.05 significance level. Sample sizes for all of the tested properties were determined according to this power analysis, except for the flexural properties for which a larger sample size of *n* = 20 was chosen to ensure a sufficiently large dataset for Weibull reliability analysis. The normality of the distribution was assessed using normal Q–Q plots and additionally formally tested using the Shapiro–Wilk test. The homogeneity of variances was tested using Levene’s test. Because no significant deviations from normality were found, parametric statistics were used for all of the analyses. All of the statistical comparisons were performed using SPSS (version 25; IBM, Armonk, NY, USA) with an overall significance level of 0.05.

Differences in MH and Ra between the initial and final values were compared with a paired *t*-test within each group. A *t*-test for independent observations was used for Δ MH, Δ Ra, and Δ E* to compare the extent of change between the C group and the IR group. In addition, the values for Δ MH, Δ Ra, and Δ E were compared among materials within each group using one-way ANOVA and Tukey’s post-hoc adjustment for multiple comparisons.

FS and FM values were compared between the C group and the IR group using a *t*-test for independent observations, while comparisons between materials within each group were performed using one-way ANOVA with Tukey’s post-hoc adjustment.

The FS values were used to perform Weibull reliability analysis by plotting the function ln [1/(1 − P_f_)] = m (ln σ − ln σ_θ_), where P_f_ = probability of failure, m = Weibull modulus, σ = flexural strength at failure, and σ_θ_ = characteristic strength [27]. The parameter “m” represents the shape parameter of the Weibull distribution and is useful for quantifying the changes in material reliability caused by exposure to radiotherapy. Weibull graphs were plotted using *n* = 20 per experimental group and a linear fit was performed using maximum likelihood estimation.

To determine differences in the average FTIR spectra between the C group and the IR group, a *t*-test for independent observations on vector-normalized spectra was performed to compare the mean absorbance values at each wavenumber. The details of the spectra comparison are described in a previous publication [28]. Differences with *p*-values less than 0.05 were considered statistically significant, i.e., indicative of a significant difference in spectral intensity at a given wavenumber. This analysis was performed using the Kinetics 1.0 add-on for Matlab (version 7.5.0, MathWorks, Natick, MA, USA).

## 3. Results and Discussion

In the present study, the effects of radiotherapy at a therapeutic dose (70 Gy over 35 cycles of 2 Gy/day) on the mechanical, chemical, and surface properties of dental restorative materials were investigated. For most of the tested properties, no statistically significant differences were found between the C group and the IR group. The only exception was color change, quantified by the ΔE* parameter, for which statistically significant effects of radiotherapy were found for one of the four materials tested. Therefore, the null hypothesis was rejected only for the color change of one material, while it was accepted for all of the other variables evaluated (MH, Ra, FS, FM, Weibull modulus, and FTIR spectra).

**Figure 1:**
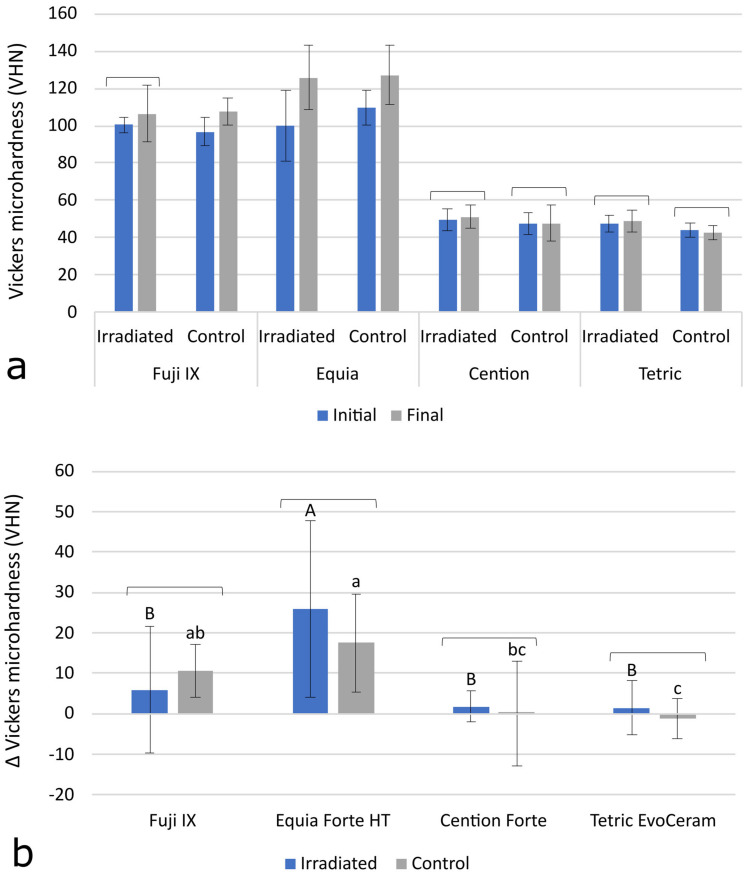
Initial and final Vickers microhardness values (**a**), and Δ microhardness values, calculated as microhardness change between initial and final values (**b**). All data are represented as mean ± 1 SD. Square brackets above bars indicate statistically similar values. The same uppercase letters denote statistically similar values within the irradiated (IR) group. The same lowercase letters denote statistically similar values within the control (C) group.

Initial and final MH data are shown in Figure 1a. A statistically significant increase in MH values over time was observed for Fuji IX (C group) and Equia Forte HT (IR group and C group) with *p*-values of 0.001 and 0.004, respectively. No significant increase in MH over time was observed for the other experimental groups. The Δ MH values in Figure 1b representing the MH change between the initial and the final measurements show no statistically significant differences between the C group and the IRm group. In the inter-material comparisons (*p* < 0.001), the significantly highest Δ MH was shown by Equia Forte HT, followed by Fuji IX. The resin-based materials Cention Forte and Tetric EvoCeram showed the lowest Δ MH values (*p* < 0.001).

Surface microhardness is a microstructural property that indicates the susceptibility of a material to permanent deformation by the indentation of a harder material. Theoretically, high-energy radiotherapy can affect the MH of resin composites, in both directions, by breaking the bonds within the polymer network (consequently reducing MH) or by enhancing the crosslinking of the polymer network (consequently improving MH) [16]. The latter effect is mediated by the increased mobility of the reactive species, which allows unreacted C=C double bonds to reach reactive sites, leading to an improvement in the degree of conversion and crosslink density [29]. This phenomenon has been described for resin composites heated after curing [30]. It is, therefore, conceivable that higher energy radiation may lead to a similar effect. This consideration is supported by the results of a study in which a linear relationship was found between radiotherapy doses in the range of 2–80 Gy and the increase in MH in resin composites [31]. As indicated by heating studies, the extent to which the degree of conversion can be improved is highly material-dependent [32], which may explain why improvements in MH due to radiation were not observed in some studies [16,19]. In cases of limited post-cure polymerization, the mechanism of bond breakage may prevail, leading to an overall decrease in MH [19]. Some authors reported an increase in MH in specimens exposed to radiotherapy but failed to identify the usual correlation between MH and the degree of conversion. Therefore, they questioned the notion that the increase in MH was due to improved polymerization and suggested that some other, unidentified structural change may be responsible for this effect [33]. Another explanation for the increase in MH due to radiotherapy was provided by de Amorim et al., who demonstrated, in a scanning electron microscopy study, that radiation can damage the resin-rich layer on the material surface, thereby exposing more inorganic filler particles and, in turn, improving MH [34]. Their study showed that the same effect can work in the opposite direction to reduce the MH of conventional glass ionomers covered with a coating resin, since in this case, the exposed underlying material is softer than the coating resin damaged by radiation. Considering other articles reporting a reduction in MH after the irradiation of glass ionomers and luting composites [19,35] and the alkasite material Cention [36], the effect of radiotherapy on the MH of restorative materials appears to be unpredictable and material-dependent. However, a review article, which included only five carefully selected studies to exclude statistical noise arising from low-level evidence, concluded that a deleterious effect of radiotherapy on MH is unlikely [20]. Our results are consistent with this conclusion, as no significant effects of radiotherapy on MH were detected for any of the materials studied. The improved MH due to the maturation of the hydrogel matrix was found for the glass-ionomer-based materials Fuji IX and Equia Forte HT; however, the extent of its improvement was statistically similar in the IR group and the C group. In contrast to the glass ionomer materials, the two resin composites tested in our study did not show significant improvements in MH over time. Despite the different extents of the increase in MH over time in the different materials, our results suggest that radiotherapy has neither a beneficial nor a detrimental effect on MH.

**Figure 2:**
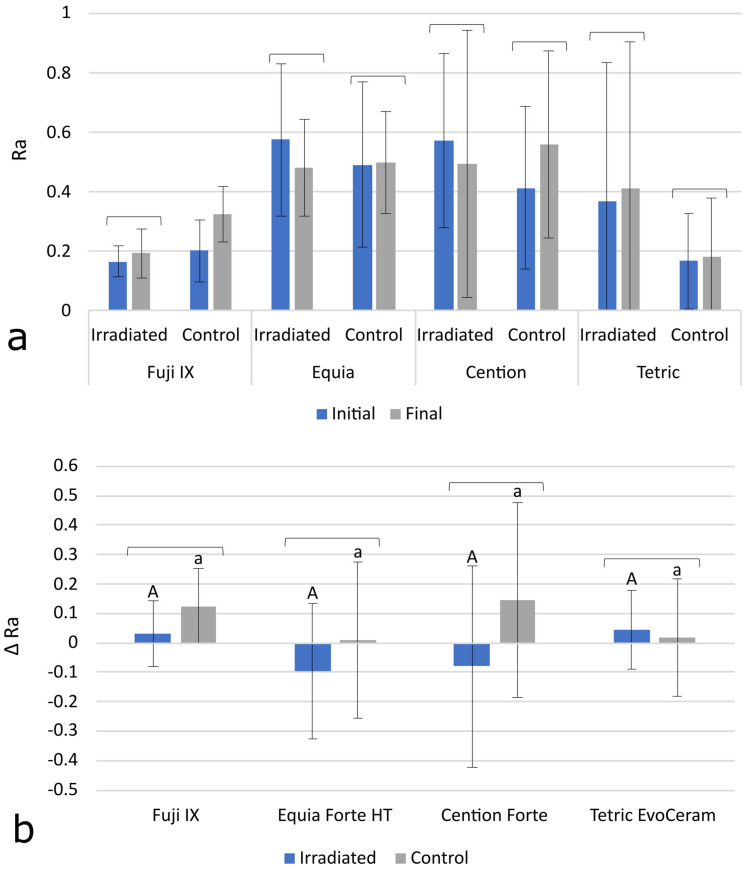
Initial and final values for the roughness parameter Ra (**a**), and Δ Ra values, calculated as Ra change between initial and final values (**b**). All data are represented as mean ± 1 SD. The square brackets above the bars indicate statistically similar values. The same uppercase letters denote statistically similar values within the irradiated (IR) group. The same lowercase letters denote statistically similar values within the control (C) group.

The initial and final values of the surface roughness parameter Ra are shown in Figure 2a. A statistically significant increase in Ra over time was found only for Fuji IX in the C group (*p* = 0.013). The results for Δ Ra in Figure 2b indicate that the changes in surface roughness over time were statistically similar between the IR group and the C group for all of the materials. There were no statistically significant differences in Δ Ra values among the materials.

The surface roughness of restorative materials is usually influenced by chemical and mechanical factors, and an increase in roughness is undesirable because it facilitates the adhesion of bacteria and the accumulation of chromogenic substances, leading to an increased risk of secondary caries and discoloration of the restoration, respectively. The potential of radiotherapy to affect the surface roughness of restorative materials is unclear, as the available literature provides mixed results. A scanning electron microscopy study by de Amorim et al., demonstrated that radiation can expose filler particles in subsurface layers of resin composites and even remove the coating resin layer of glass ionomers, resulting in surface roughening which depends on the size of the exposed particles [34]. The effect of radiation on surface roughness appears to be material-dependent, as in one study, an increase in Ra was observed only for a resin-modified glass ionomer, while no significant Ra change was observed for conventional glass ionomers and resin composites [16]. The stability of the surface roughness of resin composites exposed to radiotherapy is supported by another study that reported an unchanged Ra of a micro-filled and packable composite [19]. Conflicting results were reported by Lima et al., who found a significant increase in surface roughness due to radiotherapy in composites, glass ionomers, and resin-modified glass ionomers [37]. In a study by Ugurlu et al., Ra was investigated by atomic force microscopy, and radiation had no effect on a conventional glass ionomer and a giomer; however, a significant increase in Ra was observed for ceramic- and zirconia-reinforced glass ionomers, which was explained by increased absorption of radiation by the reinforcing fillers [38]. Our results for the Ra parameter showed that the surface roughness remained statistically similar for most materials after the 35-day period, regardless of exposure to radiotherapy. Of all the experimental groups, only Fuji IX in the C group showed a slight but statistically significant increase in Ra, which may be attributed to the low ionic strength of the immersion medium (distilled water), which may have resulted in the slow dissolution of the glass ionomer. Notwithstanding this observation, no statistically significant effects of radiotherapy on surface roughness were observed in our study for any of the materials tested.

**Figure 3:**
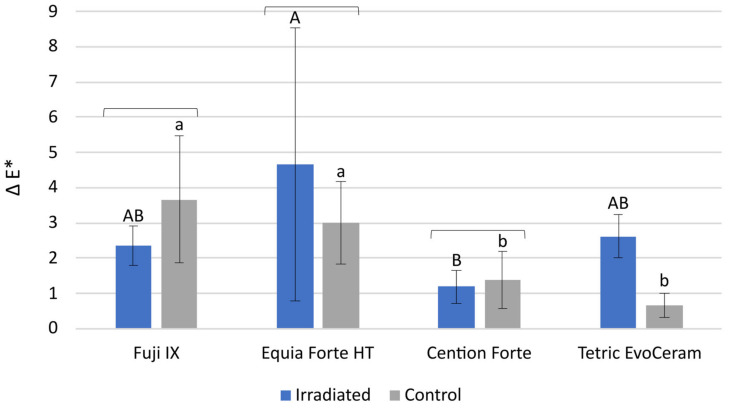
ΔE* values (mean ± 1 SD) representing color changes between the initial and final measurements. The square brackets above the bars indicate statistically similar values. The same uppercase letters denote statistically similar values within the irradiated (IR) group. The same lowercase letters denote statistically similar values within the control (C) group.

The comparison of the discoloration parameter Δ E* between the IR group and the C group indicates that the difference in perceptible color change was statistically significant only for Tetric EvoCeram which showed higher discoloration in the IR group (Figure 3), *p* < 0.001. In the comparisons among the materials (*p* < 0.001), the statistically highest Δ E* values were found for the glass-ionomer-based materials Fuji IX and Equia Forte HT, with comparatively lower values measured for the resin-based materials Cention Forte and Tetric EvoCeram.

Although the discoloration of dental restorations never leads to catastrophic failure, modern restorative materials are expected to maintain a stable aesthetic appearance throughout the service life of the restoration. Using a visible light spectrophotometer to measure the color change in the L* a* b* color space and calculate the ΔE* value as the summed color change is a convenient method to determine whether the discoloration is perceptible to the human eye. The threshold for perceptibility is usually considered to be ΔE* = 2 or, more conservatively, ΔE* = 3.3 [39,40]. Other thresholds of acceptability for ΔE* have also been suggested, ranging from 2.1 to 5.5 [25]. The effect of radiotherapy on the color change of dental restorative materials has been scarcely investigated, as there is only one study addressing this issue and reporting a significant effect for a composite, a compomer, and a resin-modified glass ionomer [18]. The authors of the study speculated that the discoloration may have resulted from the formation and entrapment of free radicals or decomposition products of phenolic stabilizers. Despite the lack of more detailed studies on the radiation-induced color change of methacrylate polymers used in dental composites, discoloration can be expected because polymeric materials are known to change color when exposed to radiation [41,42]. In our study, only the composite Tetric EvoCeram was significantly more discolored in the IR group compared to the C group, while all other materials had statistically similar ΔE* values in both groups. Overall, the higher ΔE* value for Tetric EvoCeram was the only statistically significant effect of radiotherapy observed in the present study.

Notwithstanding the fact that radiotherapy had no statistically significant effects on ΔE* for three of the four materials in our study, all of the materials tested showed some discoloration with aging. In the case of resin composites, this is due to the post-reaction slowly changing the refractive index of the polymer [43], while a similar discoloration also occurs in glass ionomers during long-term maturation [44]. Considering the conservative criterion for visually perceptible discoloration (ΔE* = 3.3), our results showed that the glass ionomer materials were close to or slightly exceeded the perceptibility threshold, indicating that their maturation-induced discoloration would be visually perceptible. Resin composites had comparatively lower ΔE* values, indicating better color stability, especially for Cention Forte, whose ΔE* values were around 1 in both groups.

**Figure 4:**
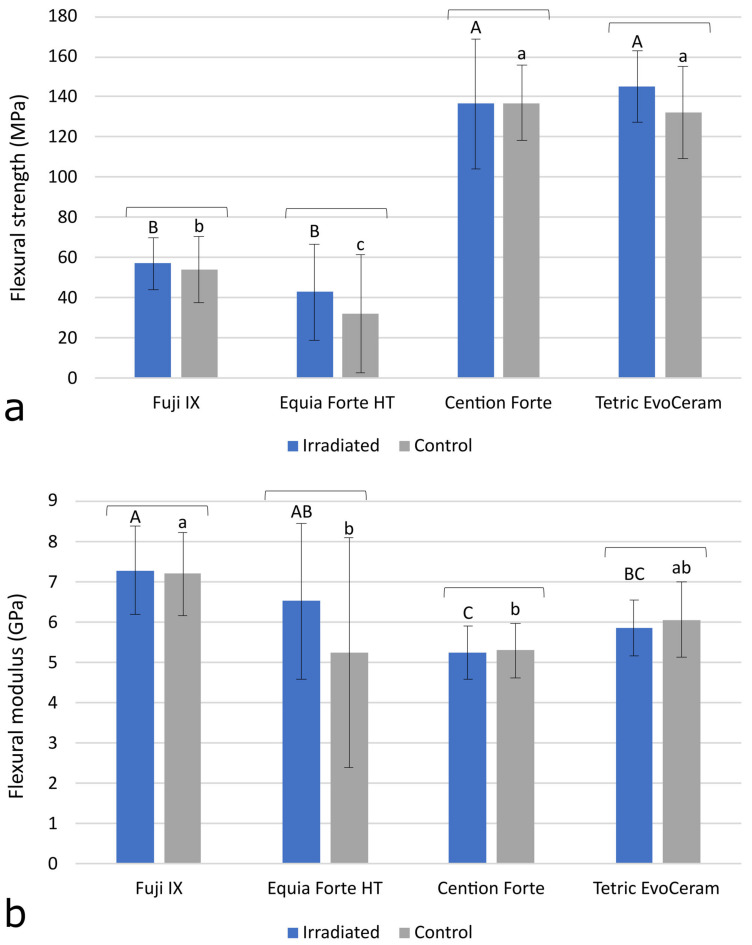
Flexural strength (**a**) and flexural modulus (**b**) measured after 35-day radiotherapy for the irradiated (IR) group or 35 days of no treatment for the control (C) group. All of the values are represented as mean ± 1 SD. The square brackets above the bars indicate statistically similar values. The same uppercase letters denote statistically similar values within the IR group. The same lowercase letters denote statistically similar values within the C group.

The results of FS and FM in Figure 4a,b respectively show no significant differences between the IR group and the C group. The glass ionomers Equia Forte HT and Fuji IX showed significantly lower FS compared to the resin-based materials Tetric EvoCeram and Cention Forte (*p* < 0.001). In contrast, FM was slightly but significantly higher for Equia Forte HT and Fuji IX compared to the resin composites (*p* = 0.001).

**Figure 5:**
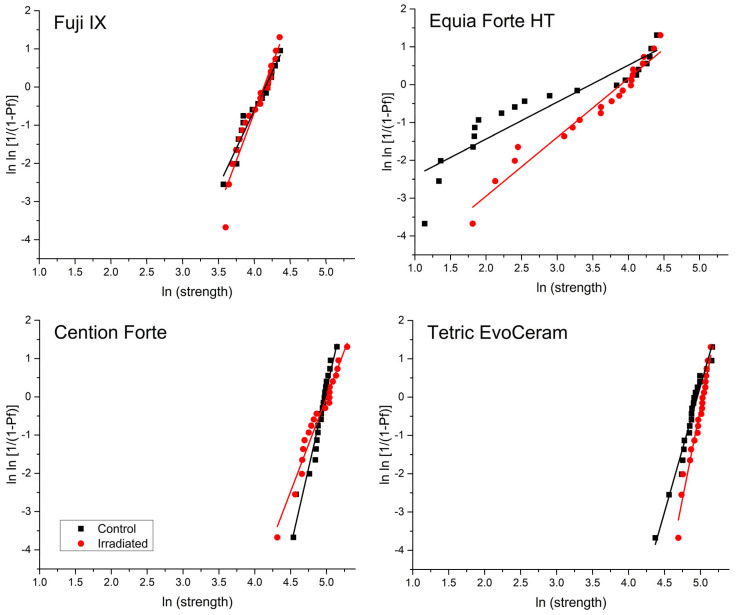
Weibull graphs for analysis of material reliability for the irradiated (IR) group and the control (C) group. The Weibull modulus is represented by the slope of the fit lines.

**Figure 6:**
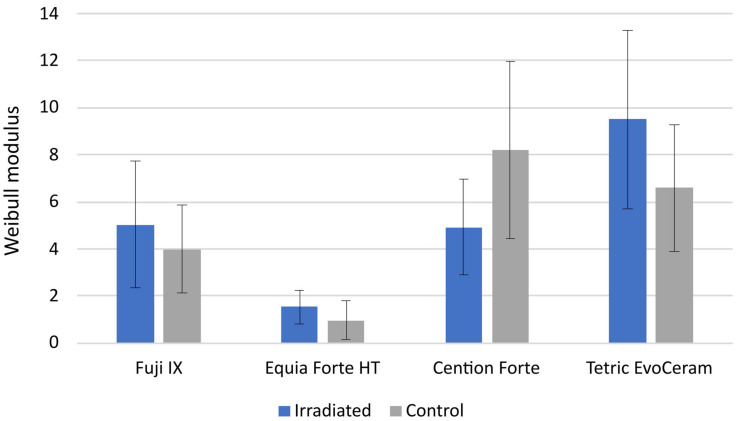
Estimated values of the Weibull modulus for the irradiated (IR) group and the control (C) group. Error bars represent limits of 95% confidence intervals.

The scatterplots and fit lines for Weibull reliability analysis are shown in Figure 5, whereas the estimated values of Weibull modulus obtained by the linear fit are presented in Figure 6. Due to considerable data variability, wide confidence intervals for the Weibull modulus values were obtained; hence, no significant difference in material reliability was found between the C group and the IR group. Overall, higher reliability values were observed for the resin-based materials Cention Forte and Tetric EvoCeram than for the glass-ionomer-based materials Fuji IX and Equia Forte HT.

FS and FM in a three-point bending test are commonly used as indicators of the macro-mechanical behavior of restorative materials. The bending test simulates a combination of tensile and compressive stresses occurring at the marginal ridge, the most sensitive part of the restoration where most fractures occur. In one study [17], FS was found to be improved by 60 Gy radiation, while in another study, the same dose reduced the FS of a composite and had no effect on a glass ionomer [35]. In two studies [13,16], a non-significant trend of FS improvement in composites by irradiation was observed, while in the latter study, a significant FS improvement was additionally observed for the conventional glass ionomer and resin-modified glass ionomer [16]. Therefore, the literature reports on the effects of FS and FM are inconclusive. In our study, no significant effects of radiotherapy were observed for FS and FM, indicating that the load-bearing capability and deformability of the material under stress were not affected by radiotherapy. In addition to analyzing the mean values of FS, a Weibull reliability analysis was performed to evaluate whether the distribution of stresses at which the materials failed was affected by radiotherapy. This analysis is performed by plotting the log-transformed values of the probability of failure (y-axis) as a function of the log-transformed values of the stress at failure (x-axis). The Weibull analysis revealed no effects of radiotherapy, which is consistent with a previous study that found no effects of radiotherapy on the Weibull modulus of resin composites, conventional glass ionomers, and resin-modified glass ionomers [16].

**Figure 7:**
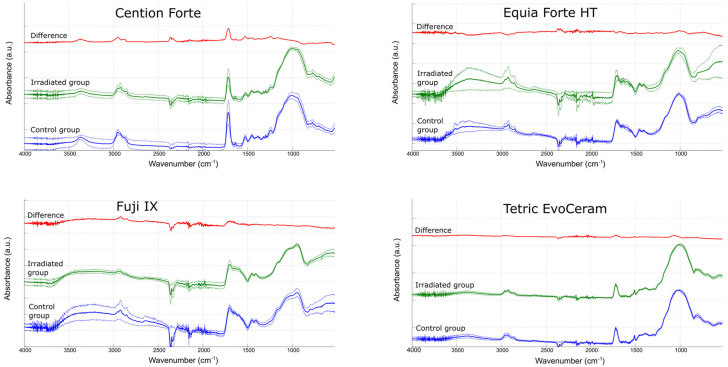
Average FTIR spectra of control specimens (blue line) and irradiated specimens (green line). The dotted lines represent ± 1 SD, while the red line represents the difference between the spectra calculated by subtracting the mean absorbance of irradiated specimens from the mean absorbance of control specimens. No differences were statistically significant.

The FITR spectra are shown in Figure 7. Although the difference between the averaged spectra of the IR group and the C group was non-zero for several spectral bands, none of these differences were statistically significant.

FTIR analysis can provide some insight into the chemical changes that result from exposure to radiotherapy, provided that these changes occur within molecular structures that give rise to FTIR spectral bands. Literature data on the effects of radiotherapy on FTIR spectra are sparse, and there are only two studies that reached conflicting conclusions [16,45]. While the study by Brandeburski et al., reported no effects of 70.2 Gy (1.8 Gy/day for 39 days) on the ATR-FTIR spectra of resin composites and glass ionomers [16], another study claimed to have found differences in FTIR spectra collected in diffuse reflectance mode for specimens irradiated with single doses of 0.25–1.00 Gy [45]. Because the results of the latter study were not supported by statistical analysis and the observed changes in the FTIR spectra were random rather than dose-dependent, the level of evidence for a deleterious effect of radiotherapy remains modest. The FTIR data from our study are also inconclusive and therefore insufficient to claim a true change in the intensity of specific spectral bands. Although the subtraction of the averaged spectrum of the IR group from that of the C group showed differences in certain spectral bands distinct from random fluctuations (e.g., the band at 1716 cm^−1^ for Cention Forte, corresponding to carbonyl group stretching), these differences were not statistically significant because of the high variability within each group. Thus, our FTIR study suggests possible spectral changes due to radiotherapy; however, the available results are not sufficient to confirm a statistically significant effect.

Since the post-reactions in restorative materials (methacrylate polymerization in composites and acid–base reactions in glass ionomers) continue for several weeks after the initial macroscopic setting, it is known that the mechanical properties, such as MH, FS, and FM, show a gradual time-dependent improvement. Depending on the experimental conditions, measurement method, and individual material composition, these improvements can be observed over several weeks or even months, but most clinically relevant improvements occur within 24 h of the initial setting [46]. As mentioned above, delivering energy from external sources has been shown to accelerate and increase the final extent of a post-cure response. This is particularly pronounced in resin composites, where polymerization is hindered by diffusion limitations in the reactive medium due to an immensely increased viscosity, which can be partially overcome by heating, allowing further polymerization. However, in the present study, no effect of the additional energy of radiotherapy was observed, since none of the post-reaction-dependent properties were significantly improved in the IR group. Since there is an upper limit to the extent of the post-reaction, it is still possible that some improvements occurred during the 35-day radiotherapy period but leveled off before the last time point, at which the final measurements were made. It is, therefore, possible that the post-reaction-dependent properties in the IR group improved in the short term but were later masked by the post-reaction in the C group, which was slower but eventually reached the same extent that was determined by the material composition, independent of radiotherapy.

In general, the radiotherapy-induced changes in the various properties of restorative materials result from a combination of “destructive” processes, e.g., chain scission caused by high-energy [47] or “constructive” processes, and the improvement of the degree of conversion and crosslinking [48], also induced by high-energy photons. It is likely that the inconsistencies in the literature on the effects of radiotherapy on restorative materials are due to differences in the relative magnitude of these two effects which affect material properties in opposite directions. Since only the combined net effect can be measured experimentally, the relative contributions of “destructive” and “constructive” processes are difficult to separate, and it is virtually impossible to predict which of the two processes will dominate in a given material without empirical testing. In this context, the finding that most materials and properties did not change in our study, as well as in other studies, does not necessarily mean that there is no effect, but rather that the two opposing processes balanced each other out, resulting in no overall measurable change.

## 4. Conclusions

Within the limitations of this in vitro study, it can be concluded that the recently introduced alkasite and glass hybrid materials were not affected by a therapeutic dose of radiotherapy. No negative effects on microhardness, surface roughness, flexural strength, flexural modulus, and material reliability were observed for the alkasite (Cention Forte) and glass hybrid (Equia Forte HT) materials or two conventional reference materials (Tetric EvoCeram and Fuji IX). The only statistically significant effect of radiotherapy was more pronounced discoloration observed for the conventional resin composite Tetric EvoCeram. It can be concluded that the deterioration of material properties in patients undergoing head and neck radiotherapy is unlikely to contribute to restoration failure.

## Figures and Tables

**Table 1:** Manufacturers’ information on the tested restorative materials.

Material (Manufacturer)	Type	Composition	LOT No.
Fuji IX (GC, Tokyo, Japan)	Conventional glass ionomer	Powder: fluor aluminosilicate glass Liquid: polybasic carboxylic acid and water	210304A
Equia Forte HT (GC, Tokyo, Japan)	Glass hybrid	Powder: fluor aluminosilicate glass, polyacrylic acid, and iron oxide Liquid: polybasic carboxylic acid and water	210201A
Cention Forte (Ivoclar Vivadent, Schaan, Lichtenstein)	“Alkasite” (resin composite with reactive glass fillers)	Powder: barium aluminum silicate glass, ytterbium trifluoride, pre-polymerized filler, calcium barium aluminum fluorosilicate glass, and calcium fluoro-silicate glass Liquid: urethane dimethacrylate, tricyclodecandimethanol dimethacrylate, tetramethyl-xylylene, diurethane dimethacrylate, polyethylene glycol 400, dimethacrylate, and Ivocerin	Z00HCG
Tetric EvoCeram (Ivoclar Vivadent, Schaan, Lichtenstein)	Conventional resin composite	Inorganic filler: ytterbium trifluoride, barium glass, ytterbium trifluoride, and mixed oxide Organic matrix: UDMA, Bis-GMA, and Bis-EMA	Z01V79

UDMA: urethane dimethacrylate; Bis-GMA: bisphenol A-glycidyl methacrylate; Bis-EMA: ethoxylated bisphenol A dimethacrylate.

## Data Availability

Data are available from the corresponding author upon reasonable request.
